# Carbon Dioxide Sensor Module Based on NDIR Technology

**DOI:** 10.3390/mi12070845

**Published:** 2021-07-20

**Authors:** Libing Zhou, Yaoyi He, Qing Zhang, Lei Zhang

**Affiliations:** 1 Tiandi (Changzhou) Automation Co., Ltd., Changzhou 213015, China; 15295023477@126.com (L.Z.); hyy@cari.com.cn (Y.H.); zhqcailiao@126.com (Q.Z.); 2 CCTEG Changzhou Research Institute, Changzhou 213015, China; 3 Science and Technology on Electronic Test and Measurement Laboratory, North University of China, Taiyuan 030051, China

**Keywords:** CO_2_, pyroelectric, lithium tantalate, the least square method, compensation algorithm

## Abstract

In this paper, a gas detection system with an environmental compensation algorithm based on nondispersive infrared (NDIR) technology was designed. The prepared infrared pyroelectric detector was a dual-channel type based on the lithium tantalate (LiTaO_3_) wafer. The design of the optical gas chamber adopted a combination of two ellipsoids and a spherical top surface, which not only enhanced the coupling efficiency of the light propagation but also facilitated the miniaturization of the sensor module. In addition to this, a temperature and humidity compensation algorithm based on the least square method was proposed to make the measurement accuracy up to ±0.9% full scale (FS).

## 1. Introduction

In recent years, with the development of industry, the problems of air pollution and global warming are becoming increasingly serious, and the gas monitoring system has obtained a sufficient market for development. CO_2_ gas sensors are essential in the field of gas detection, for purposes such as underground gas monitoring, high-temperature fire detection, and air quality detection [1,2]. Infrared gas sensors based on nondispersive infrared absorption spectroscopy (NDIR) not only overcome the shortcomings of gas sensors based on catalytic or electrochemical principles, i.e., prone to poisoning, aging, and a short life, but also have high detection accuracy, a large range, high reliability, a long service life and other recognized advantages, which makes this a research hotspot and development direction in the future [2,3,4,5]. Faced with the rapid development of the Internet of Things, new gas sensors with miniaturization, high detection accuracy, and intelligence are urgently needed for both military and civilian use. Compared with metal oxide ceramic PZT [6,7,8,9] and BST [10], single-crystal material lithium tantalate generally has better pyroelectric properties. For example, lithium tantalate has the characteristics of a large pyroelectric coefficient, high Curie temperature, high detectivity, and small dielectric constant [11,12,13]. It is an ideal material for preparing pyroelectric sensors, so it has been widely used in pyroelectric detectors. Wang et al. from Jilin University designed and used a rotating system based on a stepping motor and a single reflector spherical lens to realize and enhance the detection of multiple gases, including CH_4_, CO_2_, and CO [14]. However, the most interesting factor is that the gas sensor is easily affected by environmental factors, such as temperature and humidity, so it should have the advantages of better anti-interference ability and a stable and reliable system [15,16,17]. Traditional sensors or detection systems mostly adopt hardware compensation. Generally, the temperature compensation is carried out by the negative feedback of the op-amp, but the compensation effect of the hardware circuit is not ideal, and the temperature drift also exists in the components of the feedback circuit. Therefore, researchers have begun to study the software compensation of gas sensors.

Thermopile CO_2_ sensors developed by Seunghwan Yi et al. at the National Jiaotong University of Korea include a special purpose integrated circuit (ASIC) chip for signal regulation, a temperature sensor, and a unique dual elliptical optical waveguide. By combining the relationship between the temperature and the concentration, a general equation can be obtained, based on which the concentration of the CO_2_ gas can be estimated to be between 253 and 333 K. It has a good linear relationship with the actual concentration, with a maximum deviation of 5% [18]. Tan et al. from the North University of China proposed a miniaturized and integrated multi-gas detection system, which used the environmental compensation method to ensure that the three gases could be accurately measured under the conditions of changing temperature, humidity, and pressure [19]. Bossche et al. showed that sensor accuracy could likely be improved by optimizing the voltage regulator that powers the gas sensor’s heater, and by measuring and compensating for the difference in the partial oxygen pressure of the air that was sampled during calibration and validation experiments [20]. Grangeat et al. proposed an innovative linear quadratic model of the optical measurement and a fluidic model to compute the transcutaneous carbon dioxide pressure and the arterial blood pressure from the NDIR measurement. Additionally, preprocessing of the measured signal has been introduced [21]. Pierre et al. developed a new wearable wristband that measures the carbon dioxide gas released through the skin as a result of local heating. The nonlinear phenomena observed on the calibration curve are well described by using a linear quadratic model with non-integer powers, and the influence of disturbing gases, such as water vapor, is considered [22].

This paper introduces a pyroelectric detector based on the lithium tantalate wafer and a miniature gas sensor system. The functional relationship between the gas concentration and the electrical signal was obtained through calibration experiments of standard CO_2_ gas. On this basis, using the least square method under the condition of a fixed environmental variable, the curve compensation equations of temperature and humidity were solved, respectively. In the final experimental verification, it was proven that the temperature and humidity compensation scheme could effectively reduce the measurement error, and the accuracy could reach ±0.9% FS at 5%.

## 2. Theory

The sensor in this paper was developed based on the absorption characteristics of the infrared spectrum. When infrared radiation passes through gas molecules with spectrum absorption characteristics, the gas molecules will absorb part of the infrared radiation. This absorption relationship conforms to the Lambert–Beer law, as follows [23,24]:

(1) $$I=I_{0}\times \exp(-\varepsilon cL)$$

where *c* represents the concentration of the gas to be measured in the gas chamber; *ε* represents the absorption coefficient of the gas, which is related to the wavelength of infrared light; *L* represents the optical path of the infrared absorption; and *I*_0_ and *I* represent the intensity of infrared radiation before and after passing through the target gas, respectively. However, this law only holds for monochromatic light. Here, 4.26 μm represents the absorption wavelength of CO_2_, and 3.95 μm represents the wavelength at which the gas absorbs weakly or does not absorb, so it can be approximated as *ε*(*λ*_2_) = 0 [19,25,26]. Before the infrared radiation reaches the surface of the detector, the filter filters out any infrared light other than the targeted wavelength. Therefore, the infrared light reaching the measurement channel decreases with the increase in the CO_2_ concentration, while the infrared light reaching the reference channel hardly changes with the change in the CO_2_ concentration. This is because there are not only other disturbances in the gas chamber, but also the responsiveness of the sensitive element to different wavelengths is different. Thus, the Lambert–Beer law can be modified to obtain the electrical signal, as follows:

(2) $$U(\lambda_{1})=I_{0}(\lambda_{1})A(\lambda_{1})B(\lambda_{1})\cdot \exp(-\varepsilon (\lambda_{1})cL+D(\lambda_{1}))\;U(\lambda_{2})=I_{0}(\lambda_{2})A(\lambda_{2})B(\lambda_{2})\cdot \exp(-\varepsilon (\lambda_{2})cL+D(\lambda_{2}))$$

where *A*(*λ*_1_) and *A*(*λ*_2_) represent the responsiveness of the sensitive element to two wavelengths; *B*(*λ*_1_) and *B*(*λ*_2_) represent the coupling parameters of the optical system; *D*(*λ*_1_) and *D*(*λ*_2_) represent interference factors of the measuring system. By solving the above two equations, we can obtain:

(3) $$C=\frac{1}{L[\varepsilon (\lambda_{1})-\varepsilon (\lambda_{2})]}\{-\ln\frac{I_{0}(\lambda_{1})A(\lambda_{1})B(\lambda_{1})}{I_{0}(\lambda_{2})A(\lambda_{2})B(\lambda_{2})}+\ln\frac{U(\lambda_{1})}{U(\lambda_{2})}+[D(\lambda_{1})-D(\lambda_{2})]\}$$

Since the two channels are in the same gas chamber, the infrared radiation passes through the CO_2_ gas at the same time, so it can be approximated as *D*(*λ*_1_) = *D*(*λ*_2_).

(4) $$k_{0}=\frac{I_{0}(\lambda_{1})A(\lambda_{1})B(\lambda_{1})}{I_{0}(\lambda_{2})A(\lambda_{2})B(\lambda_{2})}$$

Finally, the above equation can be simplified as:

(5) $$C=\frac{1}{L\cdot \varepsilon (\lambda_{1})}\cdot [\ln\frac{U(\lambda_{1})}{U(\lambda_{2})}-\ln(k_{0})]$$

It can be seen that in the measurement, after the infrared radiation with wavelengths *λ*_1_ and *λ*_2_ passes through the gas cell, the ratio of the output signal between the reference channel and the measurement channel is not associated with factors such as light source fluctuations, light path interference, and environmental errors.

## 3. Design and Fabrication of Sensor Module

### 3.1. Preparation and Integration of Detector

**Figure 1:**
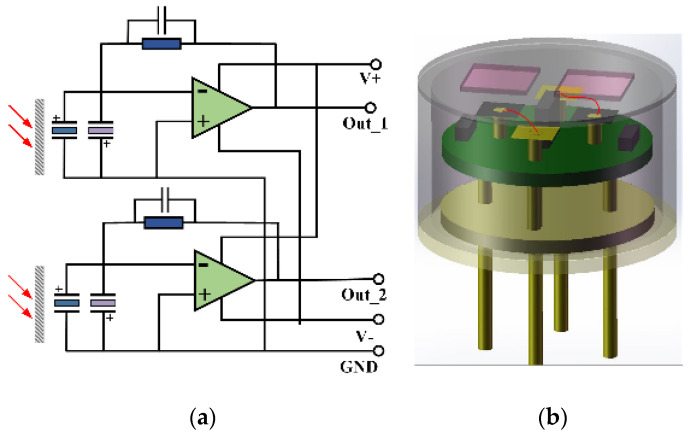
(**a**) Principal diagram of the sensor. (**b**) 3D schematic diagram.

As a typical pyroelectric material, the thickness and roughness of lithium tantalate have a great influence on the responsiveness and detectivity of the detector. The smaller the thickness of the wafer, the lower the thermal mass, and the higher the responsiveness of the detector. In addition, when the surface roughness of the wafer is smaller, it shows a smaller dielectric loss and lateral thermal conductivity, which is beneficial as it improves the detectivity. The thickness of initial lithium tantalate wafers was about 200 μm, and they must be ground to 20 μm to meet the requirements. As the key part of the sensitive element, the infrared absorption layer needs to meet three conditions: (1) a high absorption rate, (2) a low heat capacity and high thermal conductivity, and (3) strong adhesion and stability [6,27]. Using a thermal resistance coating machine as the thermal evaporator, first, the vacuum chamber was pumped to a vacuum state as low as 1 Pa by a mechanical pump; then, nitrogen with a pressure of 10^4^ Pa was injected for about 10 min to eliminate oxygen; finally, the nitrogen pressure was adjusted to 3.5 × 10^3^ Pa. In this nitrogen atmosphere, the gold sample and 502 glue were evaporated together. This method did not affect the infrared absorption and helped to enhance the adhesion between the gold-black layer and LiTaO_3_ to prevent them from falling off. Selecting the metal pillar to support the sensitive element not only ensured that the electrode of the sensitive element was effectively grounded, but also helped to reduce the heat loss of the sensitive element, thereby improving the responsibility of the detector. The main problems of pyroelectric detectors based on lithium tantalate are as follows: (1) lithium tantalate is also a piezoelectric material, which is more sensitive to vibration and stress; (2) the temperature and light in the environment will also interfere with the output signal. Therefore, the sensitive element growing with the gold-black layer and the sensitive element that does not grow with the gold-black layer are in reverse parallel to offset the superposition interference caused by the above two factors. As mentioned in chapter 2, in order to eliminate the errors caused by many factors and improve the measurement accuracy of the sensor module, we adopted the dual-channel (measurement/reference) design mode. Figure 1 shows the principal diagram and 3D schematic diagram of the detector. The preamplifier circuit is essentially an I-V (current–voltage) conversion circuit, so the operational amplifier is used to constitute the conversion circuit. In the schematic: the operational amplifier AD8627 has low power consumption, rail to rail input characteristics, and an extremely low input bias current of 1 pA; feedback resistance is 100 GΩ; partial pressure resistance is 47 KΩ; the feedback capacitance is in the order of pF. The detector was prepared according to the following detailed steps: (1) Connection and fixation of the sensitive element: first, the electrodes of the two sensitive elements were connected with ultrasonic gold wire pressure welding technology, and the electrode leads were connected with the welding pad on the positive surface of the PCB board; second, the sensitive element was bonded to the metal pillar with a low-temperature silver paste, and they were dried and solidified; third, the metal pillar was fixed to the hole of the PCB with conductive silver paste. (2) Preparation of PCB board: the front side of the PCB board was arranged with wiring and resistance, and the back side was arranged with capacitance and an operational amplifier. (3) Packaging: the TO-5 type shell was selected, and the cap and seat were aligned and welded in a vacuum environment.

### 3.2. Optical Gas Chamber

**Figure 2:**
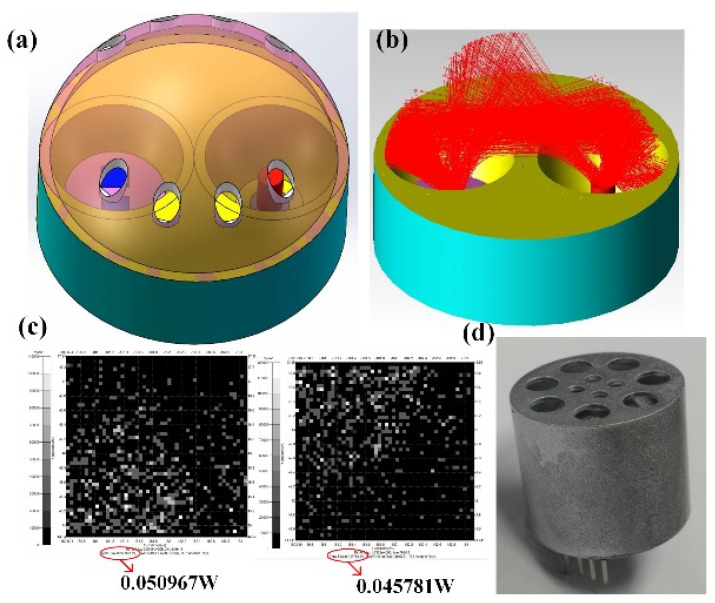
(**a**) 3D structure diagram of the optical chamber; (**b**) tracing simulation diagram of the absorbed rays; (**c**) comparison of luminous fluxes absorbed by two sensitive elements; (**d**) actual sample diagram of the gas sensor module.

As shown in Figure 2a, the optical gas chamber is composed of a spherical top surface and two hollow semi-ellipsoids. After a precise size calculation, the upper surface of the detector is located at the focal point of the ellipsoid. The detector and the infrared light source are symmetrically installed around the center point, and the spherical top surface has a good reflection and convergence effect so that the rays can be well converged to the sensitive element of the detector. As can be seen in Figure 2b, the rays were concentrated on the detector surface after two or three surface reflections, and the absorbed rays were mainly distributed in a certain direction. As the rays were reflected two or three times, this greatly increased the optical length. We punched holes in the spherical top surface in the other direction to ensure that the gas entered the gas chamber quickly, and at the same time, it could also avoid the leakage of rays. The optical software TracePro was used to simulate the rays’ traces in the gas chamber. In the simulation, we set the reflectivity of the gas chamber surface after electroplating to be 95%; the luminous flux of the light source was 1 W, with a total of 10,000 rays; the effective absorption angle of the sensitive element of the detector was −38.5–38.5°. As can be seen in Figure 2c, the luminous fluxes absorbed by the two sensitive elements were nearly equal, i.e., 0.05 and 0.045 W, respectively, which better ensured the consistency of the luminous flux reaching the two sensitive elements. By tracing and calculating the absorbed rays, the optical path of the absorbed rays could be obtained, and most of the optical path values were distributed between 41 and 43 mm, which not only ensured the response intensity of the detector but also improved the sensitivity of the detector. Figure 2d is the actual sample diagram of the microgas chamber, which is composed of two ellipsoids, a spherical top surface, a protective shell, and a post-processing circuit.

### 3.3. Detection System and Software

**Figure 3:**
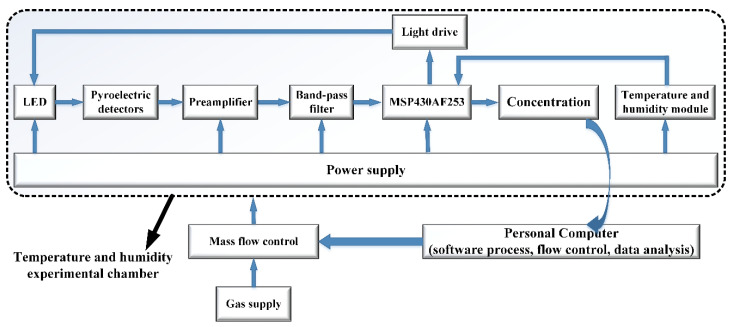
The system framework of the gas sensor module.

The relevant measurements and calibrations of the whole gas sensor module were carried out in the temperature and humidity experimental cabinet. The mass and flow control method were used to control the gas concentration input into the experimental cabinet through the PC software. The power supply module mainly includes: a 5–3.3 V voltage conversion circuit used to supply power to the MCU; 5–(−5) V voltage conversion circuit used to supply power to operational amplifier AD8627; 5–3 V voltage conversion circuit used to supply power to the light source driving module FDC6420C; 5–2.5 V voltage conversion circuit used to provide a reference voltage for the AD acquisition of the MCU. The detector’s original output current signals ranged from several pA to one hundred pA, far greater than the AD8627’s input bias current of 1 pA. The AD8627 is a low-power operational amplifier with a rail-to-rail output. A dual-power I-V converter is used for preamplification, which can convert the pA-level original signal output by the detector to voltage and amplify it to the mV level for subsequent filtering and amplification. The dual power band-pass filter removed the interference of the preamplified signal and amplified the useful signal again. MSP430AFE253 was chosen as the control core MCU, which has 24-bit high-precision ADC, a differential function, and an ultra-low power consumption. It was used to collect and process the voltage signal from the detector, the temperature and humidity information from the temperature and humidity module SHT20, to control the light source driver module to modulate the infrared light source, and finally to calculate the final gas concentration according to the compensation algorithm. The system framework is shown in Figure 3.

**Figure 4:**
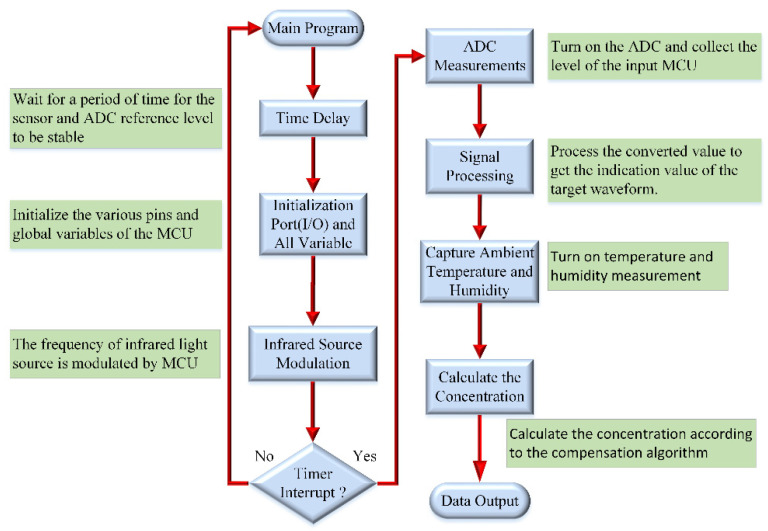
Flowchart of the program design of the sensor module.

The experimental results show that the infrared absorbance not only varies with the gas concentration but is also related to the ambient temperature, humidity, and signal capture. At the same time, the infrared light source also needs to be modulated by the microprocessor. Figure 4 shows the flow chart of the program design.

## 4. Method of Calibration and Compensation

**Figure 5:**
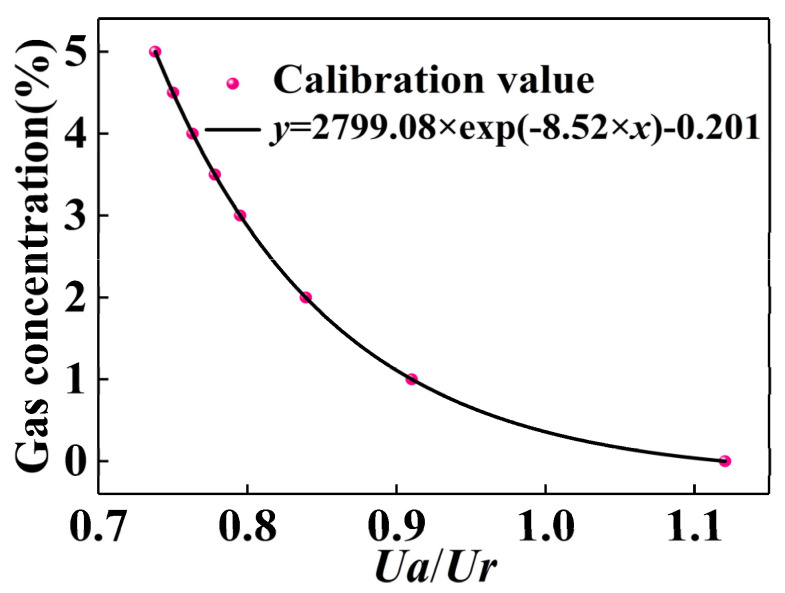
Fitting relationship between gas concentration and voltage ratio.

The environment in the experiment cabinet was set to a temperature of 20 °C and a humidity of 50%. The gas in the standard gas cylinder was configured into standard carbon dioxide gas with different concentrations through the gas distribution instrument, that is, 0, 1, 2, 3, 3.5, 4, 4.5, and 5%, respectively. Then, they were introduced into the experiment cabinet. For the accuracy of the measurement, first, we ensured the airtightness of the experiment cabinet was satisfactory; second, when the gas concentration reached the set value, we measured and recorded the voltage ratio of the two channels after stabilizing for 5 min. Then, the corresponding relationship between the concentration of CO_2_ and the ratio of the voltage was obtained through data fitting. The measurement results are shown in Table 1, and the corresponding function fitting results are shown in Figure 5. By fitting the data with an exponential function, we can obtain: y=a·exp(−bx)+c, where *a* = 2799.08, *b* = 8.520, *c* = −2.01, and *x* is the ratio of the measurement channel to the reference channel. It can be found that the ratio of the measured channel to the reference channel decreases as the CO_2_ concentration increases.

**Figure 6:**
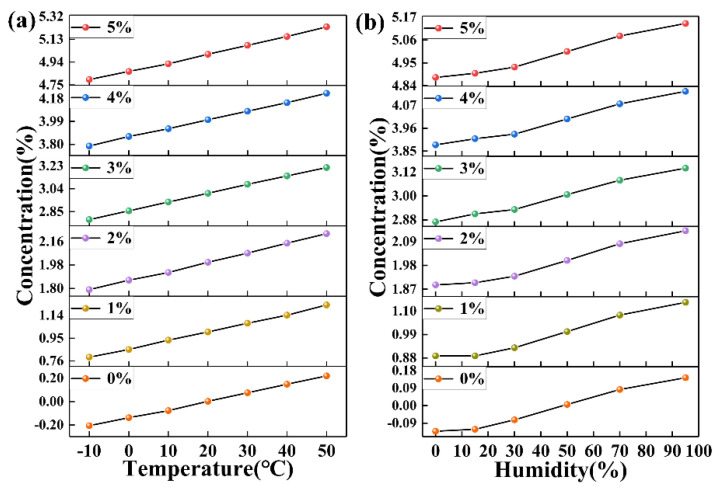
Actual concentration measurements of standard gases at (**a**) different temperatures and (**b**) humidity without concentration compensation.

The temperature and humidity of the environment will affect the accuracy of the sensor. First, we adjusted the humidity of the experimental cabinet to 50% and selected seven temperature points, and then measured the standard gas with different concentrations at different temperatures. The measurement results are shown in Figure 6a. It can be seen that the CO_2_ concentration measured by the system at 20 °C is relatively accurate. However, at other temperatures, with 20 °C as the center, the greater the temperature change, the greater the measurement error, and they are approximately linear. 

In order to compensate for the influence of temperature on the measurement value, the least square method was used to perform data fitting for the deviation value. In order to simplify the calculation, the deviation value of each temperature point was averaged to obtain ∆*C_i_*, and a cubic polynomial was used to fit:

Δ(*T*) = *aT_i_*^3^ + *bT_i_*^2^ + *cT_i_* + *e* (6)

Considering *T_i_* = −10, 0, 10, 20, 30, 40, and 50 °C, respectively, the sum of squares of deviation between the curve equation to be solved and ∆*C_i_* is:

(7) $$y=\sum_{i=0}^{5}{\left[\Delta (T)-\Delta C_{i}\right]}^{2}=\sum_{i=0}^{5}{\left(aT^{3}+bT^{2}+cT+e-\Delta C_{i}\right)}^{2}$$

(8) $$\frac{dy}{da}=2\sum_{i=0}^{5}{\left(aT^{6}+bT^{5}+cT^{4}+e-\Delta C_{i}\right)}^{2}=0$$

(9) $$\frac{dy}{db}=2\sum_{i=0}^{5}{\left(aT^{5}+bT^{4}+cT^{3}+e-\Delta C_{i}\right)}^{2}=0$$

(10) $$\frac{dy}{dc}=2\sum_{i=0}^{5}{\left(aT^{4}+bT^{3}+cT+e-\Delta C_{i}\right)}^{2}=0$$

(11) $$\frac{dy}{de}=2\sum_{i=0}^{5}{\left(aT^{3}+bT^{2}+cT+e-\Delta C_{i}\right)}^{2}=0$$

Combining the above formula, we can calculate: *a* = −8.333 × 10^−8^, *b* = 1.014 × 10^−5^, *c* = 7.02 × 10^−3^, and *e* = −0.143. Therefore, the equation of the temperature compensation curve is: ∆(*T*) = −8.333 × 10^−8^*T*^3^ + 1.014 × 10^−5^*T*^2^ + 7.02 × 10^−3^*T* − 0.143.

Similarly, the humidity compensation curve equation can be obtained as: ∆(*h*) = −2.067 × 10^−8^*h*^3^ + 2.213 × 10^−5^*h*^2^ + 9.269 × 10^−4^*h* − 0.089.

Finally, the equation of gas concentration is: C(CO_2_) = C(20 °C, 50%) − ∆(*T*) − ∆(*h*).

**Figure 7:**
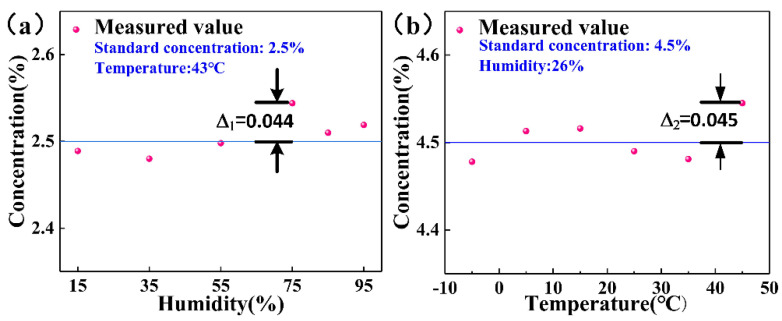
After concentration compensation, the actual measured value of standard concentration gas in different (**a**) humidity environments and (**b**) temperature.

After inputting this hybrid curve equation into the MCU, the sensor module can perform corresponding calculations and compensation according to the collected temperatures and humidity values. The temperature in the experiment cabinet was set to 43 °C, and CO_2_ gas with a standard concentration of 2.5% was introduced. Then, we changed the humidity in the experimental cabinet successively, that is, 15, 35, 55, 75, 85, and 95%, and waited for the result to tend to a stable value before recording the concentration value. The result is shown in Figure 7a, and the maximum error value is ∆_1_ = 0.044. Using the same method, the humidity in the experiment cabinet was set to 26%, and CO_2_ gas with a standard concentration of 4.5% was introduced. Then, the temperature in the experiment cabinet was changed in turn, namely, to −5, 5, 15, 25, 35, and 45 °C, and we waited for the result to stabilize before recording the concentration value. The results are shown in Figure 7b, and the maximum error value is ∆_2_ = 0.045. Therefore, the full-scale accuracy was calculated according to the following formula:

(12) $$\frac{\max|C_{m}-C_{s}|}{R}\times 100\%$$

where *C_s_* is the standard concentration value, *C_m_* is the measured concentration value, and *R* is the full-scale value. The measuring range of this system is 5%, and the maximum deviation is 0.045%, so the full range accuracy of the system is ±0.9% FS. This detection accuracy is slightly higher than that of companies such as *Korno* and *Empaer* on the market. Their products only support temperature compensation, and the detection accuracy of the high-precision type is ≤1% FS. It can be seen that after the temperature and humidity compensation, the deviation between the measured value and the standard value of the same CO_2_ concentration in different environments was significantly reduced, which improved the environmental adaptability and measurement accuracy of the system, and proves that temperature and humidity compensation is effective.

However, for infrared gas sensors, range and accuracy are contradictory and cannot be achieved at the same time. Therefore, we can only sacrifice accuracy while satisfying a large range. It can be seen from Figure 5 that the lower the concentration of CO_2_, the lower the concentration resolution will be. As a result, for CO_2_ with a concentration of only 800 ppm (the upper limit of the concentration of good air quality), there will be some deviation in the detected concentration value, which is a major limitation of the performance of the sensor module.

## 5. Conclusions

In this paper, a CO_2_ gas detection system based on nondispersive infrared spectroscopy was designed and implemented, which included a pyroelectric detector with two channels, a miniature optical chamber with a high coupling efficiency and a large range, and a circuit board. The temperature and humidity environment compensation algorithm obtained by the least square method was imported into the MCU, which ensured that the detection system could be used normally under the condition of temperature and humidity change, and the accuracy could reach 0.9% FS. Therefore, it will be widely used in the field of gas detection in industrial development.

## Figures and Tables

**Table 1:** Voltage ratio at different CO_2_ concentrations.

CO_2_(%)	*Ua*/*Ur*
0	1.120
1	0.910
2	0.839
3	0.795
3.5	0.778
4.0	0.763
4.5	0.750
5	0.738
